# Recognizing Alzheimer’s disease and Parkinson’s disease as astrogliopathies

**DOI:** 10.4103/NRR.NRR-D-25-01452

**Published:** 2026-02-05

**Authors:** Woojin Scott Kim, Onur Tanglay, YuHong Fu

**Affiliations:** Brain and Mind Centre, The University of Sydney, Sydney, NSW, Australia; School of Medical Sciences, The University of Sydney, Sydney, NSW, Australia; School of Biomedical Sciences, University of New South Wales, Sydney, NSW, Australia; Randwick Clinical Campus, School of Clinical Medicine, Faculty of Medicine and Health, University of New South Wales, Randwick, NSW, Australia

Astrocytes are the largest and most diverse glial cells in the central nervous system (CNS), with the main functions of providing support, nourishment and protection for neurons, the cells responsible for processing and transmitting information. Mounting evidence indicates that astrocytes are more than just support cells for neurons; they play complex and diverse roles that are essential for the homeostatic maintenance of the brain and spinal cord, with a key role as responders to pathological changes. As such, in recent years, there has been a dramatic increase in the interest of astrocytes in the pathogenesis of neurodegenerative diseases, Alzheimer’s disease (AD), and Parkinson’s disease (PD). Astrogliopathy is defined as pathological alterations in astrocytes, encompassing structural, molecular, or functional abnormalities that disrupt their normal roles in CNS homeostasis. These abnormalities include astrocyte reactivity, i.e. astrogliosis, degeneration, loss of supportive functions, metabolic dysregulation, impaired neurotransmitter clearance, disrupted blood-brain barrier interactions, and aberrant inflammatory signaling. Now, astrogliopathy is recognized to be a major player in both AD and PD pathogenesis. Understanding the relationship between astrocytic molecules and the accumulation of pathogenic proteins amyloid-β (Aβ) and α-synuclein will identify previously unrecognized pathways and targets, opening up new strategies for the therapeutic treatment of AD and PD, respectively.

**Versatile roles of astrocytes:** Astrocytes are a type of glial cells in the CNS that appear star-like with finely branched projections, as first described by Mihaly von Lenhossek in 1895. The past two decades of research in astrocytes have revealed a myriad of physiological functions of astrocytes. Among these are, astrocytes modify neural pathways in the adult brain by engulfing and eliminating synapses and modulating synaptic transmission and plasticity; sustain neuronal health by maintaining energy metabolism, producing trophic factors, and regulating reactive oxygen species (ROS); and participate in the neurovascular unit, exchanging small molecules with the peripheral circulation, and clearing toxic solutes via the glymphatic pathway with aquaporin-4 (Wang et al., 2025). As such, astrocytes are pivotal in supporting neuronal functions and protecting neurons from various insults. Reactive astrocytes demonstrate versatility in response to local CNS and peripheral signals, taking on neuroprotective or neurotoxic states. Accordingly, reactive astrocytes can either positively or negatively modulate the progression of CNS disorders. Reactive astrocytes are dichotomized into “A1” and “A2” subtypes according to their role in mediating the neuro-immune axis. A1 astrocytes are thought to enhance the accumulation of Aβ via mitogen-activated protein kinase 10, MAPT (tau protein), and TMED10 (transmembrane p24 trafficking protein 10), which are all differentially expressed in this subtype in AD (Ilgun and Cakir, 2025). A2 astrocytes are immunoregulatory and can promote neuronal survival, outgrowth, synaptogenesis and phagocytosis in ischemia. Conversely, RNA sequencing in aged mouse brains of A1 astrocytes revealed a pro-inflammatory phenotype with reduced homeostatic functions. Indeed, A1 astrocytes are more abundant in AD and PD brains, compared to age-matched healthy controls.

Senescent astrocytes share some characteristics with reactive astrocytes, such as the expression of inflammatory markers; however, this permanent state of astrocytes is characterized by distinct transcriptomic changes. A recent study comparing the transcriptome of reactive and senescent astrocytes found that the genes associated with neurodegeneration (DKK1, NPTX1, and CYR61) and matricellular proteins (CYR61, THBS1, and CTGF) were upregulated in senescent status (Simmnacher et al., 2020). The authors linked senescence to mammalian target of rapamycin and DNA damage responses signaling pathways, and reported that senescent astrocytes exhibit a proinflammatory status with downregulated cholesterol synthesis. Interestingly, the most prominent transcriptional changes demarcating astrocytic senescence in humans were found in the hippocampus and substantia nigra, the two stereotypical brain regions that are vulnerable to AD and PD, respectively. Astrocyte activity may therefore be crucial to maintaining cognition and motor control in healthy ageing. This was evident in a study demonstrating an association between preservation of spatial learning and memory in ageing cognitively unimpaired rats, and upregulation of hippocampal astrocytic metabolism. Elimination of senescent astrocytes, using senolytics or reprogramming astrocytes towards rejuvenation, could provide therapeutic potential in neurodegenerative diseases.

**Figure 1:**
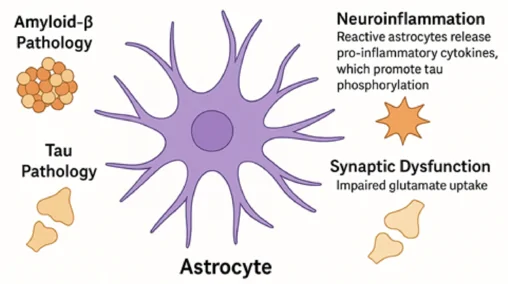
Roles of astrocytes in Alzheimer’s disease pathogenesis.

**Role of astrocytes in Alzheimer’s disease:** AD is pathologically characterized by neuronal production of Aβ and formation of Aβ plaques extracellularly. Increasing evidence indicates that dysfunction of astrocytes contributes to neurodegeneration in AD (**Figure 1**). In many clinical trials of AD, drugs that target neurons and Aβ have failed to provide clinical benefit to AD patients. One possible reason could be due to the lack of consideration of the contribution of astrocytes to the neurodegeneration processes underlying AD. To address this, much recent research has focused on understanding the relationship between astrocytes and AD pathology. Apart from the well-established astrocytic response to Aβ aggregation, a body of evidence suggests that astrocytes could be partaking in the initial process of Aβ pathology. A proteomic analysis of more than 2000 healthy and diseased brains revealed that altered astrocyte and microglial metabolism was most strongly associated with AD and occurred early in the disease. These proteomic changes most likely reflect the initial neuroprotective glial response and highlight the pivotal role of glia in AD. The involvement of astrocytes in Aβ pathology comes from the fact that reactive astrocytes display increased levels of amyloid precursor protein, β-secretase, and γ-secretase, all of which are required for Aβ production. An age-dependent upregulation of amyloid precursor protein in astrocytes was also observed in mice preceding Aβ plaque deposition. In a large mapping study to determine astrocytic changes along the spatiotemporal progression of AD neuropathology, it was found that a trophic factor-rich subcluster of astrocytes declined along pathology stages, whereas another subcluster increased in the late stage but returned to baseline levels in the end stage of the disease, suggesting an exhausted response with chronic exposure to neuropathology (Serrano-Pozo et al., 2024).

Neuroinflammatory signals can drive astrocytes to increase Aβ production, and astrocyte-derived apolipoprotein E (ApoE) influences Aβ aggregation, transport, and clearance. Astrocytes may worsen neuronal injury by releasing extracellular vesicles containing Aβ that trigger neuronal death. Conversely, they are also important for Aβ clearance. Astrocytic low-density lipoprotein receptor-related protein helps remove Aβ by regulating Aβ-degrading enzymes and intracellular degradation pathways, but low-density lipoprotein receptor-related protein expression is reduced in AD brains. Co-culture studies show that astrocytes and microglia collaborate to clear Aβ, supported by cytokine-mediated signaling and tunneling nanotubes that enhance Aβ uptake and degradation. Boosting astrocyte phagocytic capacity could represent a therapeutic strategy for AD.

Aβ induces a loss of mitochondrial potential in astrocytes but not in neurons. Astrocytes demonstrate greater reliance on glycolytic metabolism compared to neurons, and inhibition of glycolysis causes Aβ accumulation within and around astrocytes. Human induced pluripotent stem cell-derived astrocytes with PSEN1 mutations switch astrocyte metabolism to oxidative phosphorylation, resulting in increased ROS but reduced lactate production. Although the importance of the astrocyte-neuron lactate shuttle hypothesis remains controversial, it raises the potential of impaired neuronal energy homeostasis. Astrocytes in 5×FAD transgenic mice also displayed altered levels of tricarboxylic acid cycle metabolites, and these changes were induced in wild-type astrocytes by exogenous Aβ. In human astrocytes, exposure to Aβ initially resulted in increased mitochondrial fusion, with eventual mitochondrial swelling, suggesting disrupted energy metabolism in AD brains (Zyśk et al., 2023). Moreover, astrocytes take up toxic fatty acids released by hyperactive neurons and metabolize them via mitochondrial β-oxidation. Impaired mitochondrial function may consequently disrupt this process in AD and manifest in cytotoxicity. Since the transport of fatty acids is ApoE-dependent, ApoE polymorphisms may also alter the astrocytic capacity to eliminate neuronal lipids. ApoE4 astrocytes displayed impaired mitochondrial fission and mitophagy (Schmukler et al., 2020), resulting in reduced astrocytic phagocytosis of Aβ.

**Role of astrocytes in Parkinson’s disease:** PD is the second-most common neurodegenerative disease, primarily characterized by the formation of neuronal Lewy bodies, consisting of α-synuclein deposits, and degeneration of dopaminergic neurons in the substantia nigra pars compacta. A substantial number of studies investigating the role of astrocytes in PD has emerged in recent years, with a particular focus on α-synuclein, neuroinflammation, oxidative stress, NLRP3 inflammasome, extracellular vesicles, and signaling pathways (Chen et al., 2025). α-Synuclein deposits are present in astrocytes, and as astrocytes do not self-generate α-synuclein, the astrocytic α-synuclein deposits are likely to be due to uptake of extracellular α-synuclein. In PD, astrocytic α-synucleinopathy is correlated with the severity of loss in nigral dopaminergic neurons. *In vitro* studies have shown cell-to-cell spread of pathologic α-synuclein. Neuron-derived exosomes were found to transfer toxic forms of α-synuclein between neurons and astrocytes. The uptake of extracellular α-synuclein causes changes in gene expression profile, inflammatory responses, and damage to lysosomes and mitochondria. Once the phagocytic potential is depleted, astrocyte function may be altered, converting from a neuroprotective to a neurotoxic state, with subsequent prion-like spread of α-synuclein (Liddelow et al., 2017). Stressed astrocytes send out tunnelling nanotubes, enabling intercellular transfer of molecules, which may be a potent way for the spread of α-synuclein from astrocytes to other cells. The mutant SNCA (A53T and A30P) can trigger endoplasmic reticulum stress in astrocytes and reduce the levels of glial-derived neurotrophic factor in neuron-astrocyte co-culture. These studies collectively indicate cellular machinery in astrocytes respond differently to the early and late pathological forms of α-synuclein.

Oxidative stress is a well-recognized factor in the pathogenesis of PD. Midbrain astrocytes in PD patients demonstrate a greater expression of heme oxygenase-1, a stress protein that promotes iron deposition and mitochondrial dysfunction. Astrocytic antioxidant mechanisms are also dysregulated in PD. DJ-1 deficient astrocytes show reduced Nrf2 transcription activity, an antioxidant transcriptional factor that confers neuroprotection in mouse models of PD. Together, these observations suggest that increased ROS production and dysregulation of antioxidant mechanisms lead to increased oxidative stress in PD. Enhancing these antioxidant pathways may therefore hold therapeutic potential. Mitochondrial function is intricately linked with mediating oxidative stress, inflammation, and calcium and energy metabolism. Both neuronal and astrocytic mitochondrial dysfunction have been implicated in PD (Bantle et al., 2021), and targeting astrocyte mitochondria in treating PD may alleviate the deleterious effects of neuroinflammation and promote neuroprotective astrocyte functions.

Neuroinflammation plays an important role in PD pathogenesis, in which astrocytes are involved. Higher concentrations of oligomerized α-synuclein upregulated NLRP3, caspase-1, and interleukin-1β expression in mouse astrocytes. The substantia nigra has among the highest levels of TLR4 expression in the brain. A prolonged exposure of a co-culture system to physiological levels of α-synuclein oligomers led to TLR-4 sensitization in human astrocytes with consequent TNF-α and ROS production. Astrocytic TLR-4 sensitization could therefore be among the initiators of neuronal damage in PD and may contribute to the conversion of senescent astrocytes from neuroprotective to pro-inflammatory. Glycoprotein non-metastatic melanoma protein B (GPNMB) may exert its neuroprotective effect by reducing astrocyte-mediated neuroinflammation in a CD44-dependent manner. Overexpression of GPNMB in mice attenuated MPTP (1-methyl-4-phenyl-1,2,3,6-tetrahydropyridine)-indued neuron loss, and reduced gliosis and inflammation (Budge et al., 2020). Apart from releasing pro-inflammatory molecules, astrocytes promote inflammation by acting as antigen presenting cells in the PD brain to activate T-cells (Rostami et al., 2020). The same study also demonstrated intercellular transfer of α-synuclein/major histocompatibility complex-II complexes between astrocytes via tunnelling nanotubes, which may induce other astrocytes to become antigen presenting cells. Additionally, octadecaneuropeptide, a diazepam-binding inhibitor-derived peptide expressed by astrocytes, was found protecting midbrain dopamine neurons from MPTP-induced degeneration in mice via downregulation of neuroinflammatory, oxidative, and apoptotic processes. Furthermore, vitamin D-activating enzyme CYP271 expressing astrocytes appeared in PD patients, with implied neuroprotective functions by sequestering the toxic form of the α-synuclein (Mazzetti et al., 2022). These reports suggest the potential of modulating astrocytes to inhibit the detrimental effect of neuroinflammation in PD.

Apart from neuroinflammation, astrocytes interact with other CNS cells in PD that produce multiple and diverse effects. These include reducing metabolic and trophic support to neurons; failing to clear glutamate and toxic proteins; disrupting oligodendrocyte function and myelin integrity, and disturbing axonal conduction in basal ganglia circuits; and mislocalized or dysfunctional aquaporin-4 reduces waste clearance, contributing to protein aggregation and neuronal stress. In terms of endothelial cells and blood–brain barrier, astrocyte pathology causes reduced expression of tight-junction supporting factors, impaired regulation of blood flow and glucose transport, and greater infiltration of peripheral immune cells, which further activates microglia and astrocytes.

**Limitations:** Current animal and *in vitro* models provide valuable insights into astrocyte biology, but they do not fully replicate human-specific features and disease chronology. Mouse astrocytes differ noticeably from human astrocytes in size, complexity, gene expression, and functional diversity, limiting translational relevance. *In vitro* systems, including induced pluripotent stem cell-derived astrocytes, often exhibit immature phenotypes and lack the multicellular interactions and dynamic microenvironment present in the brain. Both animal and *in vitro* models do not replicate the decades-long progression and ageing-related changes seen in humans. Furthermore, most models oversimplify pathology by focusing on single genetic drivers and cannot capture polygenic, epigenetic, and environmental influences. These limitations hamper accurate modeling of astrocyte heterogeneity, reactivity, and chronological changes critical for understanding AD and PD.

**Conclusion:** Astrocytes, the most abundant and functionally diverse glial cells in the CNS, are now recognized as active contributors to neurodegenerative diseases rather than passive neuronal support cells. Astrogliopathy – comprising reactive changes, degeneration, metabolic disruptions, impaired neurotransmitter clearance, altered blood-brain barrier interactions, and abnormal inflammatory signaling – plays a central role in the pathogenesis of AD and PD. By influencing the handling of key pathogenic proteins such as Aβ and α-synuclein, astrocytic dysfunction helps drive disease progression, and understanding these processes may reveal new therapeutic targets.
